# Remote (250 km) Fiber Bragg Grating Multiplexing System

**DOI:** 10.3390/s110908711

**Published:** 2011-09-08

**Authors:** Montserrat Fernandez-Vallejo, Sergio Rota-Rodrigo, Manuel Lopez-Amo

**Affiliations:** Department of Electric and Electronic Engineering, Public University of Navarra, 31006 Pamplona, Spain; E-Mails: sergio291085@hotmail.com (S.R.-R.); mla@unavarra.es (M.L.-A.)

**Keywords:** sensor multiplexing, remote sensing, Raman amplification, fiber Bragg gratings (FBGs)

## Abstract

We propose and demonstrate two ultra-long range fiber Bragg grating (FBG) sensor interrogation systems. In the first approach four FBGs are located 200 km from the monitoring station and a signal to noise ratio of 20 dB is obtained. The second improved version is able to detect the four multiplexed FBGs placed 250 km away, offering a signal to noise ratio of 6–8 dB. Consequently, this last system represents the longest range FBG sensor system reported so far that includes fiber sensor multiplexing capability. Both simple systems are based on a wavelength swept laser to scan the reflection spectra of the FBGs, and they are composed by two identical-lengths optical paths: the first one intended to launch the amplified laser signal by means of Raman amplification and the other one is employed to guide the reflection signal to the reception system.

## 1. Introduction

Remote sensing has received increased attention in recent years due to the fact that it has proven to be a useful tool for monitoring a wide range of parameters in many fields. In general, the pivotal idea behind Remote Sensing is the continuous monitoring of structures from a central station located tens or hundreds of kilometers away from the field through the critical location of sensors which send information to the central location. This remote capability allows immediate damage detection and consequently necessary actions can be quickly taken. Furthermore, this strategy removes the logistical inconvenience of electrical power feeds to remote locations [1–3].

The more powerful remote sensing systems can find important applications in structural monitoring of large infrastructure components, such as oil or gas pipelines, ultralong bridges and tunnels, river banks and offshore platforms [1,4]. There are other promising applications of remote sensing to be highlighted. Firstly, tsunami detection and warning before their arrival to the coast, which is intended to mitigate as far as possible the disasters [5,6]; secondly, geodynamical monitoring such as surveillance of volcanic and tectonic areas which is used to predict the possible evolution towards critical stages or to detect landslides [7]; and finally, railway applications like train speed measurement, derailment, wheel defects and rail crack detection, to name but a few. Methods currently in use suffer from complexity and slow response times [8]. Optical systems, nevertheless, are very hopeful and offer very high accuracy and the possibility of real time measurement. These potential practical applications are the justification of this growing interest.

Among the wide variety of available sensors, both optical and non-optical, Fiber Bragg Gratings (FBGs) are the strong candidates for this kind of systems due to the interesting advantages they offer. They present resistance in hostile environments, good linearity, simple demodulation concepts, electromagnetic immunity, compactness, embedding capability, commercial availability and low cost. On top of that, one of the major advantages can be attributed to their wavelength-encoded information, thus the information remains immune to power fluctuations along the optical path. Another attractive benefit is their high multiplexing capability. These inherent characteristics make them attractive for applications in harsh environments and smart structures [9].

Another two important issues must be taken into consideration when remote sensing systems are designed. Firstly, the interrogation system, and secondly, the most suitable amplification method must be chosen to compensate for the losses undergone by the light.

Some methods have been proposed and reported in the scientific literature with two essential motivations: to increase the number of sensors multiplexed in a single network and to enable extending the distance while maintaining a good signal to noise ratio [10]. The first systems were based on broadband light sources in which case the maximum distance was limited to a maximum of 25 km, mainly due to Rayleigh Scattering [11]. In order to surpass this limit, FBG sensors systems which are composed by an in fiber linear cavity laser scheme are a promising option. These systems usually include Raman amplification [2], or Raman amplification merged with other kinds of amplification: Brillouin, Erbium doped fiber or both [12–14]. To the best of our knowledge the longest distance covered by a FBG sensor system for a single FBG reported to date reached 230 km, with a signal to noise ratio of 4 dB [3]. Following these approaches this research field is being extensively investigated at present.

In this work, we propose and demonstrate two ultra-long range fiber Bragg grating sensor systems based on the utilization of different fibers to send and to collect the optical signals from sensors. In real applications, the cost of the system does not have a significant added increment if two single mode fibers (SMF) are used inside the fiberoptic cable instead of only one. On top of that, this kind of scheme with two optical paths becomes easy-going sensor systems which address some of the dominating limitation factors such as double Rayleigh scattering.

In our first approach four FBGs are located 200 km from the monitoring station and a signal to noise ratio of 20 dB is reached. Finally, an improved version is able to detect the FBGs placed 250 km away with a signal to noise of 6–8 dB. Both systems are based on a wavelength swept laser to scan the reflection spectra of the FBGs.

## 2. Description of the Remote Sensing System

**Figure 1.**
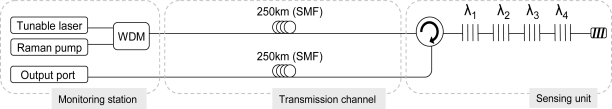
Schematic depiction of the ultra-long fiber Bragg grating sensor system.

The basic design of our simple 200 and 250 km ultra-long fiber Bragg grating sensor systems is schematically depicted in Figure 1.

As it is shown in Figure 1 the system could be divided into three essential sections. The sensor unit is composed by four multiplexed FBG sensors and a circulator which redirects the reflected signals towards the output port. The FBGs are disposed in serial configuration and located within the Raman-amplified wavelength band. Their central wavelengths are λ1 = 1,555.24 nm, λ2 = 1,549.82 nm, λ3 = 1,546.88 and λ4 = 1,552.40 nm, each one showing a bandwidth of 0.19 nm, 0.16 nm, 0.19 nm and 0.24 nm and a reflectivity of 98.9%, 98.3%, 99% and 99.8% respectively. Initially, this serial configuration could seem an obstacle to achieve power equalization for the channels, however, in this scheme it is not a problem, because the system is not based on a long distance laser structure. In those systems [2], the mode competition has crucial influence when all the channels must lase at the same time. In our proposed system, the channels equalization depends on both the non-uniform shape of the Raman profile and the insertion loss of the FBGs located in front of the sensor interrogated in each moment.

**Figure 2.**
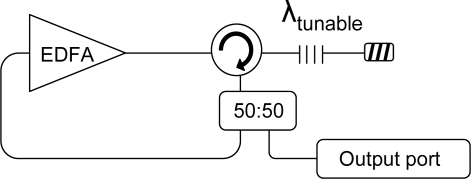
Basic design of the tunable laser.

As far as the monitoring station is concerned, it is comprised by a wavelength division multiplexer (WDM) which combines the signal which comes from the tunable laser and the Raman pump. The fiber Raman laser emitting at 1,445 nm is deployed to generate distributed Raman amplification in the system. On the other hand, two different tunable lasers are utilized depending on the length of the sensor system when the network reaches 200 km a commercial ANDO tunable laser with a bandwidth of 100 MHz is used to sweep the whole span, but when the FBGs are located at a distance of 250 km from the monitoring station some nonlinear effects appear. Namely, stimulated Brillouin scattering (SBS) arises with the adverse effect that this entails. A detailed analysis of this issue will be presented in the next section. Consequently, a tunable laser with a wider bandwidth is deployed to cope with these impairments. The tunable laser is based on a previously studied and published scheme by the authors [15]. The basic design of the laser, which included a tunable FBG to confer it tunable capability is depicted in Figure 2. The tunable FBG has a bandwidth of 0.6 nm and offers an extinction ratio of 65 dB. It is able to sweep the whole span.

On the other hand, the monitoring station also includes the detection system, which in our case is a commercial OSA (Advantest Q8384). Finally, the transmission channel consists of two identical-length optical paths. The first one intended to launch the amplified laser signal by means of discrete Raman amplification and the other one is employed to guide the reflection signal to the reception system.

The justification of using two paths, which doubles the needed fiber in the system, is based on the reduction of the effective cost of fiber optic components, especially SMF cables. Furthermore, in real applications the final cost of the installed system is not significantly increased if two fibers are used instead of only one.

The operation mode of the remote sensing system is the simplest one. To interrogate the remote fibre Bragg gratings sensors, the tunable laser makes a wavelength sweep of the band where the FBGs are located, avoiding the utilization of modulated signals, as in [3]. We also demonstrate the benefits of avoiding the utilization of high coherence length lasers, as employed in other remote systems with Rayleigh scattering limitations [16,17].

## 3. Experimental Results and Discussion

The experimental results for ultra long-range FBG interrogation systems are summarized in this section:

### 3.1. Remote Sensing (200 km) FBG Interrogation System

**Figure 3.**
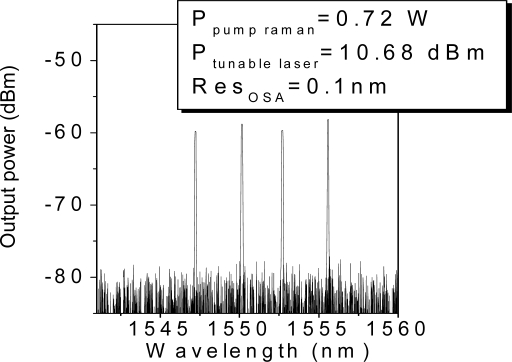
Spectrum of the reflected signal from the four remotely multiplexed FBGs.

**Figure 4.**
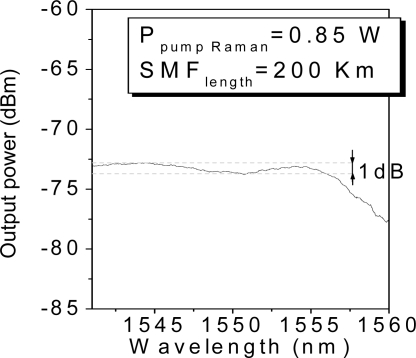
Amplified spontaneous emission at 200 km.

In our experiment, we examine the interrogation of the sensor unit composed by four FBGs located 200 km from the monitoring station. Figure 3 shows the reflected signal when 0.72 W of Raman pump laser and 10.68 dBm of tunable laser are launched into the system. This figure collects (superposes) the individual measurements carried out when the laser wavelength is tuned into each wavelength and an additional one when the tunable laser wavelength does not fit the wavelength of any FBG, in order to estimate the noise floor. From Figure 3 we can draw some conclusions as follows: firstly, the optical signal to noise ratios (OSNR) from the four FBG remotely multiplexed varies from 20 dBm, in the worst case, to 22 dBm in the best one. As discussed in the previous section, the OSNRs are determined by the non-uniform shape of the Raman gain profile. Figure 4 shows this spectrum when the transmission channel is 200 km length. It reveals that even if the Raman gain profile is not completely uniform, the wavelength bandwidth where the FBGs are located has 1 dB of maximum deviation. As a result, the OSNR maximum variation is 2 dB between the best and the worst case. Secondly, the remote system is a low noise configuration because it copes with the two principal dominating sources of noise of a fiber Raman amplifier. The amplified spontaneous emission (ASE) generated by spontaneous Raman scattering is addressed by the FBGs, since they only reflect the Bragg wavelength. Furthermore, the multipath interference (MPI) noise mainly produced by Rayleigh backscattering (RB) does not play a crucial role because the reflected signal travels through a different optical path than the launched tunable laser and the distributed Raman amplification.

The proposed remote sensing system is a low noise configuration wherein the background noise is limited by the noise imposed by the OSA. This great signal to noise ratio encourages increasing the number of sensors to be multiplexed or to try to reach further distances. The ability to multiplex several sensors is not only relevant from the conceptual point of view, but also important for practical reasons considering that it allows, in general, a reduction in the complexity and cost the sensing system [9]. Nevertheless, a detailed discussion of this point would go beyond the scope of this paper. Thus, the next subsection is focused on locating the sensing unit further and further away from the monitoring station.

### 3.2. Remote Sensing (250 km) FBG Interrogation System

**Figure 5.**
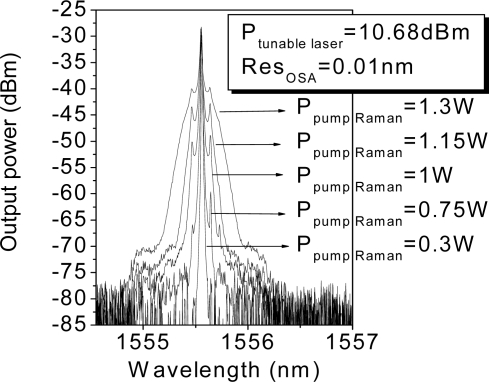
Spectrum of the tunable laser after 250 km length transmission.

**Figure 6.**
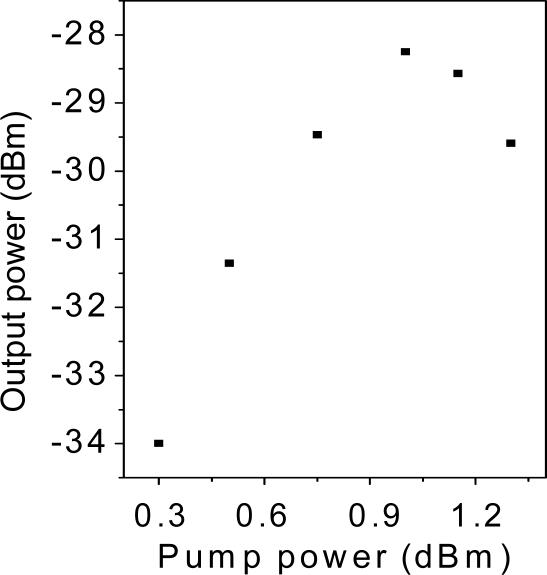
Evolution of laser power *vs.* Raman pump laser.

The first attempt to reach 250 km used the same system. It is obvious that a higher amount of Raman pump power is necessary since the amount of losses to compensate with the distributed Raman amplification is also higher than in the previous system. To this end, the Raman pump power was increased, but unsurprisingly Brillouin scattering arises, which hampers the signal amplification, as in many fiber communication systems [18]. Figure 5 shows the spectrum of the tunable laser after 250 km length of SMF, it illustrates the progression of the Stokes lines: the higher the pump power, the greater the Stokes lines power and spectrum broadening is also observed. For conventional fiber the threshold power for this process is a few mW, however, the impairments start when the amplitude of the scattered wave is comparable to the signal power. The biggest problem appears in this kind of situations when the backscattered light experiences gain from the forward-propagating signal which leads to depletion of the signal power. In consequence, there is a practical limitation of the maximum possible gain, as shown in Figure 6.

**Figure 7.**
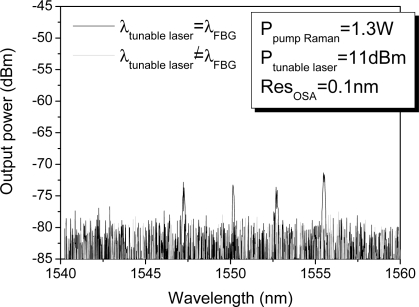
Spectrum of the reflected signal from the remotely multiplexed four FBG.

For lasers with linewidths Δλ much larger than 20 MHz, SBS gain is inversely proportional to Δλ [19]. Thus, for this 250 km length span, we have developed a tunable laser, as it is shown in Figure 2, with a wider bandwidth to reduce the problems caused by SBS. Our selected tunable laser bandwidth was 0.6 nm, and it launches 11 dBm with and extinction ratio of 65 dB. Figure 7 shows the spectrum of the reflected signal from the four FBG located 250 km away from the monitoring station. The optical signal to noise ratio is 6 dB in the worst case and 8 dB in the best one. To the best of our knowledge, this ultra-long range fiber Bragg grating (FBG) sensor system is the longest reported system and it is also worth noticing that the system is able to multiplex FBG sensors.

**Figure 8.**
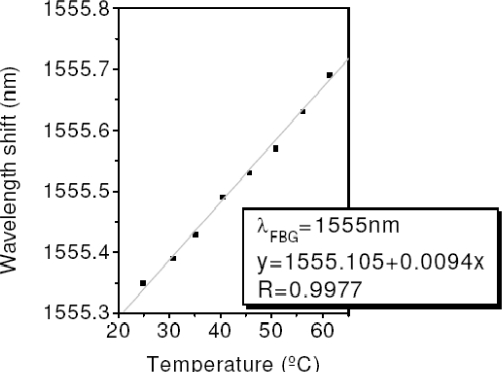
Shift wavelength of the heated up FBG.

In order to assess the sensing capability of our system, the FBG centered at 1,555 nm was located in a climatic chamber and heated up. Figure 8 illustrated the FBG linear behaviour *versus* the temperature which results in a sensitivity of 9.4 pm/°C.

**Figure 9.**
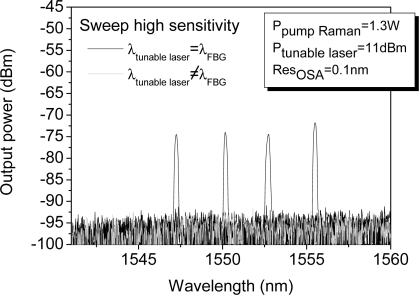
Spectrum of the reflected signal from the four FBG using OSA option sweep high sensitivity.

As mentioned previously, the proposed system is restricted by the noise level imposed by the detection scheme. In our set-up, in order to reduce this level we have used the OSA option sweep high sensitivity. This measurement option reduces the noise level averaging, thus the background noise decreases and the OSNR increases meaningfully. Thus, the measured OSNRs improve from 20 to 18 dB for the best and worst case, as Figure 9 shows.

## 4. Conclusions

We have experimentally demonstrated the feasibility of two ultra-long range fiber Bragg grating (FBG) sensor systems. Both simple systems are based on a wavelength swept laser to interrogate the multiplexed FBGs. The systems are composed by two optical paths of identical lengths: the first one launches the amplified laser signal by means of Raman amplification and the other one is employed to guide the reflection signal up to the reception system. The proposed schemes address some limiting factors such as Rayleigh backscattering. In the first approach the four FBGs were located 200 km away from the monitoring station, and a signal to noise ratio of 20 dB was reached. An optimized version of the system was able to detect the FBGs placed at 250 km, with a signal to noise ratio of 6–8 dB (18–20 dB using averaging methods). Thus, it is the longest range FBG sensor system reported to date that includes sensor multiplexing capability.
